# Pennsylvania State Veterinary Medical Association

**Published:** 1901-02

**Authors:** E. M. Ranck

**Affiliations:** Secretary


### PENNSYLVANIA STATE VETERINARY MEDICAL ASSOCIATION.

The annual meeting of this Association will convene in Philadelphia, Pa., March 5 and 6, 1901, at 3608 Pine Street.

This promises to be a meeting of profound interest, and it will be to the interest of every reader of this Journal to be present if possible.

The profession is growing every day, and these meetings help to add to its growth, not only in number, but in education.

The programme will consist of many valuable papers on all topics pertaining to veterinary medicine and hygiene as well as animal husbandry, and a special feature in the discussion of papers is about to be completed. These meetings have more than one feature to draw men together, for it is here we exchange views on different subjects, discuss subjects which may or may not be clear to ourselves, and by so doing we are benefited. Extending good-fellowship to all our professional friends, by doing this we make our own sphere more bright and pleasant. Then, from an educational stand-point, it is here we learn of things, when the facilities are greatest, that lead us to make more careful investigations, to broaden our views, to equip ourselves to be able to battle with the realities of veterinary medicine more successfully.

The local committee of arrangements will try to arrange everything to the satisfaction of all, and strangers in Philadelphia will be looked after with special care by this committee. Printed programmes will be sent to non-members who read the Journal and are interested in the welfare of Association work.

E. M. Ranck,
Secretary.

